# Bacterial Sigma Factors as Targets for Engineered or Synthetic Transcriptional Control

**DOI:** 10.3389/fbioe.2014.00033

**Published:** 2014-09-03

**Authors:** Lakshmi Tripathi, Yan Zhang, Zhanglin Lin

**Affiliations:** ^1^Department of Chemical Engineering, Tsinghua University, Beijing, China

**Keywords:** sigma factor, strain engineering, bacterial stress tolerance, extremophile, synthetic biology

## Abstract

Sigma (σ) factors are the predominant constituents of transcription regulation in bacteria. σ Factors recruit the core RNA polymerase to recognize promoters with specific DNA sequences. Recently, engineering of transcriptional regulators has become a significant tool for strain engineering. The present review summarizes the recent advances in σ factor based engineering or synthetic design. The manipulation of σ factors presents insights into the bacterial stress tolerance and metabolite productivity. We envision more synthetic design based on σ factors that can be used to tune the regulatory network of bacteria.

## Introduction

**Figure 1:**
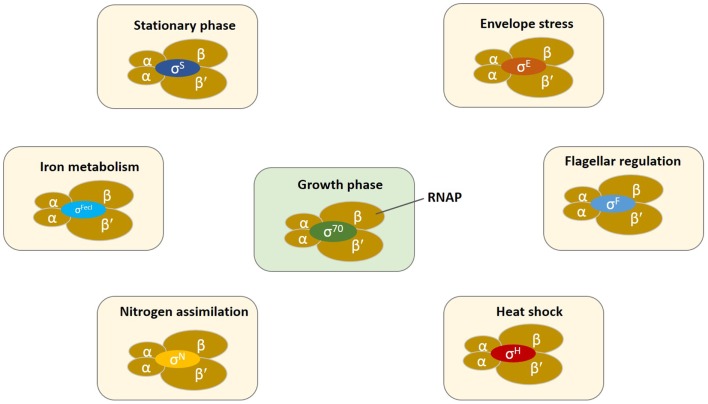
**Schematic representation of the bacterial transcription initiation in which core RNAP (α_2_ββ′ω) associates with several different classes of sigma factors for the regulation of gene expression**. Switching of σ factors occurs during the changing growth phases or environmental conditions. σ Factors in exponential growing cells, σ^70^/σ^D^/σ^A^; stationary or stress phase, σ^S^/σ^38^/σ^B^; heat shock, σ^H^/σ^32^; extracytoplasmic function or extreme heat shock, σ^E^/σ^24^; iron metabolism, σ^FecI^; nitrogen regulation, σ^N^/σ^54^; expression of flagellar genes, σ^F^/σ^28^. The small subunit ω is omitted for clarity.

The transcriptional network of bacteria contains an extensive hierarchy of regulators with the RNA polymerase (RNAP) and particularly, σ factors toward the top and other transcription factors (TF) toward the bottom that control the regulatory network of gene transcription (Ishihama, 2010). Bacterial core RNAP consists of five subunits α_2_ββ′ω with a molecular mass of ~400 kDa. σ Factors are a family of TF that recruit RNAP for the transcription of a specific subset of genes/operons (Figure 1). The core RNAP associates with the initiation σ factor and the resulting holoenzyme recognizes promoters with specific DNA motifs.

Engineering of transcriptional regulators can be utilized to modulate the bacterial transcriptional regulatory network (Lin et al., 2013b). These studies have been focused on artificial TF, such as zinc finger proteins (Park et al., 2005; Lee et al., 2011), as well as components of RNAP such as σ factors (mainly σ^70^) and the α subunit of RNAP (Alper and Stephanopoulos, 2007; Klein-Marcuschamer and Stephanopoulos, 2008; Klein-Marcuschamer et al., 2009; Ma and Yu, 2012). Recently, this approach has been further extended by introducing an engineered exogenous global regulator IrrE (Chen et al., 2011, 2012). The present review describes the recent highlights in the engineering or synthetic design of σ factors for artificial transcriptional regulation in several different types of bacteria.

## An Overview of σ Factors

Bacteria encode an essential housekeeping σ factor that controls a large number of promoters, which is also known as σ^70^ (or σ^D^) in *Escherichia coli*, σ^A^ in *Bacillus subtilis*, and other Gram-positive bacteria. While, one or more alternative σ factors control the transcriptional initiation of a subset of genes with shared functions that varies between species. For example, an intracellular pathogen *Mycoplasma genitalium* encodes 1 σ factor, *E. coli* encodes 7 σ factors, *B. subtilis* has 18 σ factors, *Pseudomonas putida* encodes 24 different σ factors, and the soil bacterium *Streptomyces coelicolor* contains 65 σ factors including 53 alternative σ factors (Gruber and Gross, 2003). All σ factors except σ^N^ (or σ^54^) belong to the σ^70^ family (Jordan et al., 2008). The σ^N^ family mainly regulates nitrogen metabolism (Murakami and Darst, 2003).

Bacteria often experience fluctuating changes in their environment from heat shock, variation in pH, and osmolarity to nutrient deprivation. They have adapted various mechanisms to respond to the imposed stresses (Aertsen and Michiels, 2004), and alternative σ factors provide the main line of response by effectively reprograming the transcription of sets of specific genes (Marles-Wright and Lewis, 2007). The key regulator of general stress response in *E. coli* is the σ factor σ^S^ (σ^38^ or RpoS) (McCann et al., 1991; Battesti et al., 2011), which either directly or indirectly regulates about 10% of *E. coli* genes during the stationary phase (Weber et al., 2005). The *rpoS* gene translation is activated by the RNA-chaperone Hfq mediated sRNAs, while it is inhibited by the sRNA, OxyS (Majdalani et al., 2002; Mandin and Gottesman, 2010). In the Gram-positive bacterium *B. subtilis*, the σ factor σ^B^ controls the general stress response (Hecker and Volker, 1998; Volker et al., 1999).

The *E. coli* heat shock response is positively controlled by σ^H^ (or σ^32^) encoded by the *rpoH* gene, which regulates the transcription of heat shock genes (Erickson and Gross, 1989; Wang and Kaguni, 1989; Nagai et al., 1990). σ^H^ mediated response protects the cells from heat as well as several other environmental stresses including acid shock, ethanol, and hyperosmotic shock (Gamer et al., 1996).

The cell surface stress is regulated by the ECF σ factors, which are in turn regulated by the corresponding anti-σ factors that are in most cases encoded within the same operons as σ factors (Missiakas et al., 1997; Ades et al., 1999). *E. coli* has two ECF σ factors, including σ^E^ (σ^24^) that regulates cell envelope response and σ^FecI^ (FecI) controlling the iron citrate transport system, respectively (Erickson and Gross, 1989; Angerer et al., 1995; Chaba et al., 2007). In *P. aeruginosa*, an ECF σ factor, AlgU (σ^22^) regulates the expression of the genes essential for synthesis of the exopolysaccharide alginate, which is responsible for biofilm formation and provides the bacterium better fitness against environmental stresses. AlgU is structurally similar to σ^E^ and recognizes similar promoter sequences (Cezairliyan and Sauer, 2009; Barchinger and Ades, 2013). *B. subtilis* encodes seven ECF σ factors (σ^M^, σ^W^, σ^X^, σ^Y^, σ^Z^, and σ^YlaC^) (Wiegert et al., 2001; Ho and Ellermeier, 2012).

A class of σ factors that deserves special attention are those of the extremophilic microorganisms, which have developed changes in the cytoplasmic membrane structure, heat shock proteins, and synthesis of extremoenzymes to live under extreme environment such as extreme temperature, pH (acid or alkaline), high pressure, high salt concentration, toxic metals, and increased radiation (Cavicchioli et al., 2011; Reed et al., 2013). The extremophilic enzymes in these organisms have received ample attention due to their potential biotechnological applications, but the regulatory proteins including σ factors are much less well understood or known.

*Thermus thermophilus* HB27 and *T. thermophilus* HB8, both are extremely thermophilic bacteria, require an optimal growth temperature of 65°C. A housekeeping σ factor, σ^A^ was found in these thermophiles, which has a similar role to σ^70^ in *E. coli* (Nishiyama et al., 1999; Sakamoto et al., 2008). *T. thermophilus* HB8 has an operon consisting of *sigE* and *TTHB212* genes encoding a σ^E^ and an anti-σ^E^, respectively (Sakamoto et al., 2008).

A homolog of the *E. coli* stationary phase σ factor σ^S^ (about 76% similarity) is found in *Vibrio parahaemolyticus*, a Gram-negative bacterium inhabiting coastal waters. The *rpoS* deletion mutant strain had significantly reduced survival under acid stress conditions, suggesting its role in cell survival under stress conditions, such as oxidative stress and exposure to acid (Whitaker et al., 2010).

The extremely radioresistant *Deinococcus radiodurans* has acquired the ability to survive acute doses of γ-irradiation. Only three predicted σ factors were found in the genome of *D. radiodurans*, including one σ^70^ (*rpoD*/*sigA*), an ECF σ ortholog Sig1, and a third putative ECF σ ortholog Sig2. Sig 1 was found to play a major role in the regulation of heat shock genes for survival against heat and ethanol stresses, while Sig2 likely controls the expression of a smaller set of heat shock genes. Other alternative σ factors such as σ^S^, σ^H^, and σ^N^ were not found in *D. radiodurans* (Schmid and Lidstrom, 2002).

The bacterial σ factors have distinct promoter targets, yet these targets sometimes overlap. ECF σ factors generally recognize the highly conserved AAC motif in the −35 region and a CGT motif in the −10 region (Helmann, 2002). For example, the ECF σ factors of *B. subtilis* including σ^W^, σ^X^, and σ^M^ recognize an overlapping set of promoters related to cell envelope homeostasis and antibiotic resistance (Mascher et al., 2007; Kingston et al., 2013). Functional overlap between the promoters of σ^70^ with those of σ^S^, σ^H^, or σ^E^ was found in *E. coli* (Tanaka et al., 1993; Wade et al., 2006). The consensus binding DNA sites for the housekeeping and ECF σ factors of *E. coli* are very different, yet ~40% of overlap was observed for promoters recognized by σ^70^ and σ^E^ (Wade et al., 2006).

## Engineering of σ Factors

Different σ factor based engineering approaches are described below and summarized in Table 1.

**Table 1:** **Various σ factor based engineering approaches applied for strain engineering**.

Sigma factor		Approach	Phenotype	Organism	Reference
Housekeeping σ factor	σ^70^	Global transcription engineering (gTME)	Ethanol, lactic acid, and acrylamide tolerance	*E. coli L. plantarum R. ruber* TH	Alper and Stephanopoulos (2007), Klein-Marcuschamer and Stephanopoulos (2008), and Ma and Yu (2012)
			Hyaluronic acid production	*E. coli*	Yu et al. (2008)
	σ^HrdB^	Random mutation, genome shuffling, point mutation	Teicoplanin production	*A. teichomyceticus* L - 27	Wang et al. (2014)
Stationary phase σ factor	σ^S^	Gene knockout	1-Propanol and putrescine production	*E. coli*	Choi et al. (2012) and Qian et al. (2009)
		Random mutagenesis	Isobutanol production	*E. coli*	Smith and Liao (2011)
		Overexpression of sRNAs	Activation of σ^S^ and increased acid tolerance	*E. coli*	Gaida et al. (2013), Bak et al. (2014), and Jin et al., 2009
		Overexpression of 5′ untranslated region of *rpoS* mRNA	Polyhydroxybutyrate (PHB) production	*E. coli*	Kang et al. (2008)
	SigE	Gene overexpression	Hydrogen and PHB production	*Synechocystis* sp.	Osanai et al. (2013a) and Osanai et al. (2013b)
Alternative σ factors	Sig6	Gene knockout	Avermectin production	*S. avermitilis*	Jiang et al. (2011)
	σ^22^	Mutation in anti-sigma factor	Alginate production	*P. aeruginosa, P. fluorescens*	Martin et al. (1993) and Borgos et al. (2013)
	Orf21	Gene overexpression	Clavulanic acid production	*S. clavuligerus* NRRL3585	Jnawali et al. (2011)
	σ^E^	Adaptive evolution, gene overexpression	Ethanol production and tolerance	*Thermoanaerobacter* sp. X514	Lin et al. (2013a,b)
	σ^N^	Gene overexpression	Oxytetracycline production	*E. coli*	Stevens et al. (2013)
σ Factors by synthetic design	σ^S^	Synthetic sRNA, construction of riboswitch	Altered *rpoS* translation	*E. coli*	Jin et al. (2013) and Jin and Huang (2011)
	ECF σ factors	Chimeric σ factors		*E. coli*	Rhodius et al. (2013)
	Orthogonal σ factors	Bisected T7 polymerase		T7 phage	Segall-Shapiro and Voigt (2013)

### Engineering of housekeeping σ factors

Global transcription engineering (gTME) was applied to *E. coli, Lactobacillus plantarum*, and *Rhodococcus ruber* TH by tailoring the housekeeping σ factor to investigate ethanol tolerance, hyaluronic acid production, lactic and inorganic acid tolerance, and acrylamide tolerance, respectively. The best σ factor mutants showed enhanced stress tolerance than the wild-type strains (Alper and Stephanopoulos, 2007; Klein-Marcuschamer and Stephanopoulos, 2008; Yu et al., 2008; Ma and Yu, 2012).

The introduction of exogenous σ factor into an industrial strain *Actinoplanes teichomyceticus* L-27 was recently demonstrated. For this strain, the exact principal sigma factors σ^HrdB^ are not well understood, but the actinomyces genera shares high similarity in σ^HrdB^ amino acid sequences. Thus, three σHrdB genes from *A. missouriensis* 431, *Micromonospora aurantiaca* ATCC27029, and *Salinispora arenicola* CNS-205 were rationally selected and engineered by random mutagenesis, DNA shuffling, and point mutation, and the resulting library transferred in *A. teichomyceticus* L-27. Screening yielded a recombinant strain with a twofold increase in teicoplanin production in a pilot scale fermentation (5.3 mg L^−1^). The mutants showed significant diversity in the region 1.1 of σ^HrdB^, apparently generated by DNA shuffling (Wösten, 1998; Campbell et al., 2002; Wang et al., 2014).

### Engineering of stationary phase σ factors

While σ^s^ mainly plays a significant role in the metabolism of *E. coli* during the stationary phase, it was reported that during the exponential growth phase, deletion of σ^s^ gene (*rpoS*) enhanced the tricarboxylic acid cycle and the glyoxylate shunt (Rahman et al., 2006). The *rpoS* gene was knocked out from a metabolically engineered *E. coli* for 1-propanol production, and the yield increased by over 100% to about 10 g L^−1^ (Choi et al., 2012). Similarly, for an *E. coli* strain producing putrescine, a four carbon linear chain diamine with a wide range of industrial applications, the deletion of *rpoS* led to a 10% increase in the productivity in both batch and fed-batch cultures (Qian et al., 2009).

An engineered *E. coli* was constructed for improved isobutanol production by random mutagenesis and selection with an analog of valine (norvaline), which theoretically stimulated the production of the common precursor 2-ketoisovalerate. The mutant strain NV3 produced an improved isobutanol level of about 8 g L^−1^ in comparison to the wild-type strain with 5 g L^−1^ in 24 h. A truncation mutation was found in the *rpoS* gene of the mutant strain. Repair of this gene further elevated the yield of isobutanol to 21.2 g L^−1^ in 99 h (Smith and Liao, 2011), likely because in this case σ^S^ was critical for isobutanol tolerance.

The 5′ untranslated region of *rpoS* mRNA forms an inhibitory loop, which blocks the ribosome binding site and represses the translation of *rpoS*. Three non-coding sRNAs (DsrA, RprA, and ArcA) can disrupt this loop and overexpression of these sRNAs increased acid tolerance supra-additively up to 8500-fold during active cell growth (Gaida et al., 2013). On the other hand, deletion of any of these sRNA decreased acid resistance (Bak et al., 2014). The sRNA GcvB also positively regulates the *rpoS* expression level. This sRNA was identified from a single gene knock out library for 79 sRNA in *E. coli* MG1655. The overexpression of *gcvB* caused a threefold increase in the *rpoS* translation. Thus, GcvB can be a potential candidate for modulating *rpoS* expression and for engineering acid tolerance (Jin et al., 2009).

A stress-induced system was designed to activate polyhydroxybutyrate (PHB) production by placing the synthesis genes under the control of the 5′ untranslated region and the promoter of *rpoS* in *E. coli*, which formed a so-called stress-induced region (SIR). The engineered strain produced PHB up to 85.8% of cell dry weight in a glucose medium without the addition of any additional inducer (Kang et al., 2008). PHB with shorter carbon chains and polyhydroxyalkanoate (PHA) with longer carbon chains are two types of well known bioplastics.

The stationary phase σ factor, SigE in cyanobacteria is a positive regulator of sugar catabolism. For the unicellular cyanobacterium, *Synechocystis* sp. PCC 6803, overexpression of *sigE* enhanced the transcription of genes for the oxidative pentose phosphate pathway and also for glycogen catabolism, and altered the metabolite levels (e.g., acetyl coA and citrate) of the TCA cycle (Osanai et al., 2011). Further studies revealed more pleiotropic effects. The cell size of *sigE* over-expressing strain increased likely due to an aberrant cell division. The hydrogen production was also increased under microoxic conditions. However, *sigE* overexpression caused reduced photosynthesis and respiration due to changes in several regulatory proteins. Regardless of these changes, the engineered strain showed normal growth and viability both under nitrogen replete and nitrogen-limiting conditions (Osanai et al., 2013a). In addition, several cyanobacteria including *Synechocystis* sp. PCC 6803 accumulate mostly PHB as carbon and energy source during unfavorable growth conditions. Under nitrogen-limiting conditions, *sigE* overexpression in *Synechocystis* 6803 increased glucose catabolism, which turned the metabolic pathway toward increased PHB production (Osanai et al., 2013b).

### Engineering of alternative σ factors

Avermectins that have potent anthelmintic and insecticidal properties are biosynthesized by *Streptomyces avermitilis*. About 47 ECF σ factors were found in the genome of *S. avermitilis*. The production of avermectin was negatively regulated by an ECF σ factor, Sig6 in *S. avermitilis* SAV663. Deletion of *sig6* resulted in an increased avermectin yield of ~680 mg L^−1^, while overexpression decreased the yield by 56–63%. The expression of a pathway-specific activator gene *aveR* was upregulated in the *sig6* deletion mutant, resulting in the increased avermectin production (Jiang et al., 2011).

Alginate is biosynthesized as an exopolysaccharide by two bacterial genera, *Pseudomonas* and *Azotobacter*. As mentioned, the expression of alginate biosynthetic genes is regulated by the alternative σ factor AlgU. A mutation in the anti-σ factor MucA resulted in stabilization of AlgU in *P. aeruginosa*, thereby overproduction of alginate (Martin et al., 1993). An efficient method for alginate production in *P. fluorescens* SBW25 was subsequently demonstrated (Borgos et al., 2013) using an engineered MucA mutant with a deletion of the 37 C-terminal amino acids. This mutant was found to upregulate various genes including the TCA cycle, ribosomal and translational proteins, and downregulated the NADPH oxidizing cycle, when cells were grown on glycerol in chemostats under nitrogen limitation.

The putative σ factor Orf21 was engineered to study its role in the production of clavulanic acid (CA) in *Streptomyces clavuligerus* NRRL3585. Disruption of the *orf21* gene decreased the CA production slightly, whereas overexpression of *orf21* enhanced the production by 1.4-fold. RT-PCR analysis showed that Orf21 activated the synthesis genes *ceas2* and *cas2*, and the activator gene *ccaR*, of the CA gene cluster (Jnawali et al., 2011).

Solvent tolerance and productivity are two important traits required for biofuel production. An adaptive evolution strategy was developed for improved ethanol tolerance and productivity in *Thermoanaerobacter* sp. X514. Long-term exposure to ethanol caused the ethanol sensitive wild-type to develop a low ethanol tolerance of 2% (X_I_) and subsequently a higher tolerance of 6% (X_II_). Genomic and transcriptomic analyses identified an iron containing alcohol dehydrogenase (ADH) and the ECF σ^24^ as the key factors for improved tolerance for X_I_ and X_II_, respectively. The *adh* over-expressing strain showed a 33% improvement in the ethanol productivity over the control, and a 31.8-fold enhanced growth under 1% ethanol. Under the same condition, ethanol tolerance was improved by 102-fold by over-expressing σ^24^, with a 21% higher ethanol productivity than the control (Lin et al., 2013a).

Overexpression of the alternative σ factor σ^54^ in *E. coli* successfully enhanced the heterologous expression of the oxytetracycline biosynthetic genes from a 32 kb type II oxytetracycline gene cluster. It was reasoned that this non-native cluster nonetheless contained σ^54^ promoters. This provides useful clues for the production of other polyketides (Stevens et al., 2013).

### Synthetic design for σ factors

So far, the σ factors have been largely manipulated with the more traditional genetic engineering approach. However, in recent years, several lines of studies using the synthetic biology approach have emerged. For example, an artificial sRNA was obtained from a rationally designed library that bound to the RBS region of the *rpoS* gene. This RNA alone or together with the sRNA DsrA can act to inhibit the translation of *rpoS* and convert the activator DsrA to a co-inhibitor (Jin et al., 2013). A theophylline dependent hybrid riboswitch was also created using the lactose inducible promoter for controlling the σ^S^ expression. When theophylline was added, the *rpoS* expression was repressed with increased acid sensitivity and increased acetate assimilation. The addition of IPTG restored the σ^S^ activity with enhanced acid survival and normal acetate assimilation (Jin and Huang, 2011).

Genome mining was used to identify an orthogonal set of 20 bacterial ECF σ factors and their cognate promoters. These σ factors and their corresponding anti-σ factors were further used to build synthetic transcriptional units in *E. coli*. Additional chimeric σ factors were obtained by swapping the −35 and −10 promoter binding domains from subgroups of ECFs. This study demonstrates the possibility of designing synthetic regulatory network within a cell by utilizing ECF σ factors (Rhodius et al., 2013).

Finally, a bisected version of the T7 polymerase was used to create orthogonal sigma factors. This split polymerase contained a larger catalytic core and a smaller segment for promoter recognition. By engineering the latter, different orthogonal promoter recognition fragments were generated (Segall-Shapiro and Voigt, 2013).

## Outlook

Although the knowledge of σ factors, including those of extremophiles, has been accumulating rapidly over the past two decades, the engineering of these factors remains scattered, and less than rational with few *a priori* designs. This is because the underlying mechanisms that bring the observed outcomes are often poorly understood. Nonetheless, the application of recent advances in systems biology will provide a more quantitative description of complex bacterial regulatory networks, which in turn will guide the engineering of σ factors to become a more predictable exercise. Additionally, an increasingly expanded set of new DNA manipulation tools as well as design principles from synthetic biology is now also available for this engineering endeavor. From this, one can envision the following lines of re-engineering or re-design of σ factors:

(1) Re-design of the different domains of σ factors, including the helix-turn-helix (HTH)-mediated DNA binding motifs. This is in part demonstrated by the pioneering work of Rhodius et al. (2013). Design of artificial transcriptional factors based on the zinc finger motifs commonly found in eukaryotes has already been proved feasible (Park et al., 2003; Khalil et al., 2012).
(2) Editing the promoter sequences for σ factors. Engineered promoters of different strength for both exponential and stationary phases have been available (Alper et al., 2005; Miksch et al., 2005), which provide clues or tools for manipulating promoters for σ factors.
(3) Rewiring of the regulatory network for σ factors, which are part of the cellular transcriptional regulatory network that is interconnected, multi-layered, and hierarchical (Martinez-Antonio and Collado-Vides, 2003). It would be interesting to see whether one could partially or completely simplify/re-design regulatory switches or circuits for σ factors in order to better respond to industrially relevant stresses, many of which are more defined than the environmental ones.
(4) Construction of sRNA molecules or sRNA circuits that regulate the translation of σ factors, as shown by Jin and Huang (2011). Na et al. (2013) have also previously demonstrated that it is possible to design artificial sRNAs that can regulate translation.
(5) Finally, utilization of extremophilic σ factors in mesophilic organisms, which has not been explored so far. Along this line, it is interesting to note that the GroESL chaperonins from the solvent-tolerant *P. putida* enhanced thermo-tolerance and ethanol tolerance in *E. coli*, and the groESL from the thermophilic *Thermoanaerobacter tengcongensis* provided better tolerance toward corn cob hydrolyzates and better productivity for *Clostridium acetobutylicum* (Luan et al., 2014). This suggests that other extremophilic proteins including regulatory proteins may function in mesophilic organisms (Lin et al., 2013b).

## Conflict of Interest Statement

The authors declare that the research was conducted in the absence of any commercial or financial relationships that could be construed as a potential conflict of interest.
